# Does the ‘right to request’ flexible work policy influence men’s and women’s uptake of flexible working and well-being: findings from the UK Household Longitudinal Study

**DOI:** 10.1136/jech-2025-224166

**Published:** 2025-09-15

**Authors:** Baowen Xue, Heejung Chung, Ran Gu, Anne McMunn

**Affiliations:** 1 Department of Epidemiology & Public Health, University College London, London, UK; 2 King's Business School & King’s Global Institute for Women’s Leadership, King’s College London, London, UK; 3 Department of Economics, City St George's, University of London, London, UK; 4 Institute for Fiscal Studies, London, UK

**Keywords:** WORKPLACE, METHODS, HEALTH POLICY

## Abstract

**Background:**

The ‘right to request’ flexible working policy has been gradually extended and, by 2014, extended to cover all workers with at least 26 weeks of continuous employment. The impact of these policy changes is unclear. This research aims to assess the effects of the 2014 policy reform on the uptake of flexible working and its impact on health and well-being, focusing on gender differences.

**Methods:**

Data were drawn from waves 2, 4, 6, 8 and 10 of the UK Household Longitudinal Study (2010–2020). We employed a doubly robust difference-in-differences method to estimate the average treatment effects on the treated of the 2014 policy reform. This analysis examined the effects on the uptake of flexible working, mental and physical health, and satisfaction with life, job and leisure.

**Findings:**

The 2014 policy reform increased women’s uptake of reduced hours work arrangements, with the effect growing stronger over time. However, no increase in uptake was observed among men. No strong effects were found for flexitime or teleworking arrangements for either men or women. Additionally, the policy reform resulted in a reduction in psychological distress and improved life satisfaction among women.

**Conclusions:**

The reduction in women’s psychological distress and improved life satisfaction might be partly explained by the increased women’s uptake of reduced hours arrangements, which may have enabled women to better meet their family care demands. However, even the gender-neutral policies on flexible working may inadvertently exacerbate gender inequalities in labour force participation by pushing women more into part-time work.

**WHAT IS ALREADY KNOWN ON THIS TOPIC**

- In the UK, the flexible working policy has been progressively expanded.
- But the impact of these policy changes is unclear.

**WHAT THIS STUDY ADDS**

- The 2014 policy reform, which removed the requirement for caring responsibilities, increased women’s uptake of reduced hours arrangements.
- It reduced women’s psychological distress and improved life satisfaction.
- However, no similar increase in uptake or well-being was observed among men.

**HOW THIS STUDY MIGHT AFFECT RESEARCH, PRACTICE OR POLICY**

- It is important to take gender into account when examining the consequences of flexible working-related policy.
- The policy should avoid inadvertently exacerbating gender inequalities in labour force participation.

## Background

**Figure 1:**
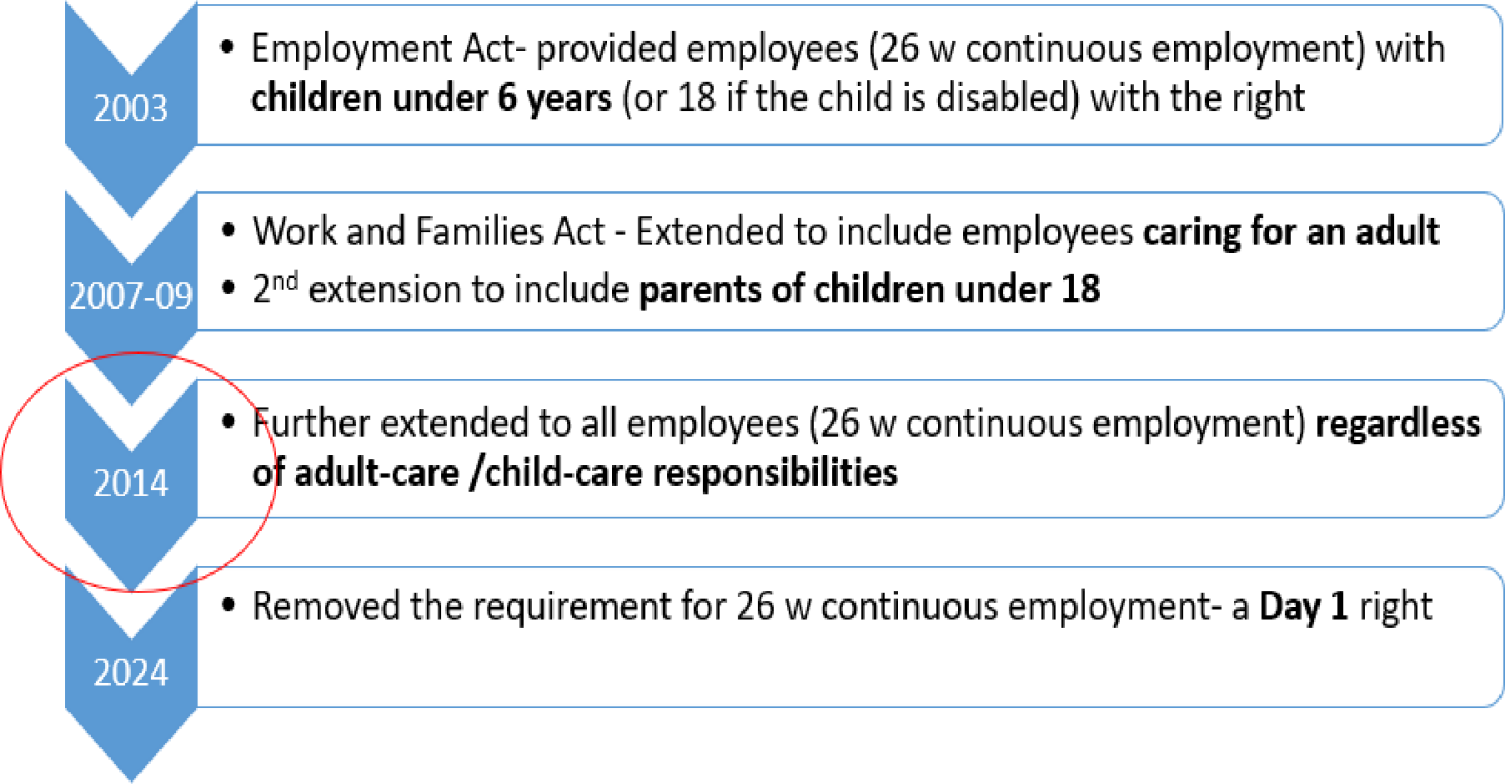
Timeline of the flexible working policy reform in the UK.

 In the UK, the ‘right to request’ flexible working policy has been progressively expanded over the past decade (see figure 1). Initially introduced under the Employment Act of 2003, this policy allowed parents of children under 6 years old to request flexible working. Eligibility was limited to employees who had been with their employer for at least 26 weeks.^1^ The Work and Families Act of 2006 broadened this right to include employees who care for a dependent adult.^1^ A further extension in 2009 expanded the rights to parents of children under 18.^2^ On 30 June 2014, the ‘right to request’ was extended once again, this time to all employees with at least 26 weeks of continuous employment, regardless of their caring responsibilities.^3^ As of 2024, all employees have the right to make a flexible working request from the first day of their employment.^4^

There are three primary types of flexible working arrangements: reduced work hours (eg, part-time work), flexible schedules and teleworking (eg, working from home).^5^ Some research has shown that users of flexible work arrangements tend to experience lower levels of work-family conflict and report better health and well-being.^6^ A 2010 systematic review concluded that self-scheduling or gradual/partial retirement is likely to improve health outcomes.^7^ However, some studies suggest that teleworking can increase feelings of work–family conflict,^8^ while other research found no association between teleworking or flexible schedules and chronic stress responses.^5^ One possible reason for the mixed results is that flexible schedules and teleworking can lead to workers working harder and longer hours.^9^ Additionally, part-time work can increase financial insecurity, which is a factor that can deteriorate workers’ mental health outcomes.^10^ A recent scoping review suggested a complex picture of the influence of flexible working and recommended more complex research designs using longitudinal data.^11^ Also, there may be gender variation in the well-being outcomes of flexible working. A study found that the availability of flexible working benefited both men and women, but actual usage only improved women’s, not men’s, mental health.^12^ This may be due to women being more likely to use reduced hours, while men are more likely to telework. For men, the stigmatised views against remote workers and the potential negative career outcomes that come with them may hinder the benefits of well-being.^13^

Despite the rapid expansion of flexible policy, the ‘right to request’ has sometimes been criticised as a ‘light-touch’ measure. While employers are required to consider such requests, they can still refuse them. Women’s ongoing roles as primary caregivers and their greater share of housework^14^ often lead them to seek flexible working arrangements as a family-friendly workplace option. But a recent survey of over 44 000 women in the British public sector shows that 30% have had their flexible working request denied.^15^ A survey in 2021 found that half of mothers were either refused their flexible working request, or it was only partially accepted.^16^

We searched for any longitudinal studies published before 6 February 2025, to find studies investigating the effect of the flexible working policy on workers’ use of flexible working and health (online supplement 1). No longitudinal studies have assessed the effects of the UK’s 2014 policy reform of flexible working, which marked a more significant step forward by removing the requirement for caring responsibilities to qualify for the ‘right to request’. Our study aims to fill this gap by evaluating both the short-term and long-term effects of the 2014 policy reform on flexible working uptake, physical and mental health, and satisfaction with life, job and leisure, for men and women separately.

## Method

### Data and sample

This research used data from the UK Household Longitudinal Study (UKHLS), also known as ‘Understanding Society’, which has encompassed around 40 000 households since 2009.^17^ As the information on flexible working and housework was measured in every other wave, we aggregated data from waves 2, 4, 6, 8 and 10, spanning from 2010/2012 to 2018/2020. We did not use data from wave 12, due to significant changes in work-from-home patterns during the COVID-19 pandemic.^18^

As the 2014 ‘right to request’ flexible working reform was effective from 30th June 2014, we consider wave 4 (interviewed between January 2012 and June 2014) as the baseline wave, and wave 6 (interviewed between January 2014 and May 2016), wave 8 (interviewed between January 2016 and May 2018) and wave 10 (interviewed between January 2018 and May 2020) as the postintervention waves. We then compared each of waves 6, 8 and 10 to wave 4 (baseline). Observations from participants interviewed between January and June 2014 in wave 6 were excluded from the data analysis to ensure that observations included in wave 6 were interviewed after the policy reform. Our eligible sample is those who were in paid employment (excluding self-employed) and had been employed for at least 26 weeks at wave 4 and had been followed up at least once at waves 6, 8 or 10. The sample size for eligible samples is 17 801. After excluding missing data, the final sample size ranges between 15 320 and 15 485, depending on the outcomes (see online supplement 2—Missing data).

### Measures

#### Control and exposure groups

The control group comprises those who are already eligible for the ‘right to request’ flexible working arrangements—those who have caring responsibilities. Based on the information collected in the UKHLS, parents of a child under 16 or being responsible for a child under 18 or being an unpaid caregiver in wave 4 were assigned to the control group. Those who were not parents or caregivers at wave 4 were assigned to the exposure group.

#### Uptake of flexible working arrangements

Employees were asked which of the following arrangements were available at their workplace and whether they currently use any of these arrangements. We grouped arrangements into three types: reduced hours arrangements (part-time, job-share and term-time working arrangements); flexitime arrangements (flexitime, annualised hours and compressed working week arrangements) and teleworking (working from home on a regular basis), with each type as a binary outcome (currently use; currently not use or not available).

#### Mental and physical health

Mental health was measured by the 12-item General Health Questionnaire (GHQ-12) and the 12-item Short Form Survey (SF-12). GHQ-12 assesses psychological distress in the general population, ranging from 0 (minimal distress) to 36 (maximal distress).^19^ The SF-12 evaluates overall health and functioning with two summary scores: Mental Component Summary (MCS) and Physical Component Summary (PCS), each with a score ranging from 0 (low functioning) to 100 (high functioning).^20^

#### Life satisfaction, job satisfaction and satisfaction with leisure

Life satisfaction was measured using a single item asking participants to rate their overall satisfaction with life on a scale from 1 (not satisfied at all) to 7 (completely satisfied). Leisure satisfaction was assessed by rating satisfaction with the amount of leisure time available, and job satisfaction was measured by overall satisfaction with the current job, each using a 1–7 scale.^21^

#### Covariates

All the covariates are measured at wave 4—the last wave collected before the policy reform. Covariates included age, ethnicity (white, black, Indian, Pakistani/Bangladeshi, other Asian/other), marital status (married, cohabiting, single, separated/widowed), highest education qualification (degree, other higher degree, A-level, General Certificate of Secondary Education (GCSE), other, no qualification), occupational class (management/professional, intermediate, routine), working hours and household income (quintiles). When assessing the mental health outcomes, SF-12 PCS was additionally adjusted. When assessing the SF-12 PCS outcome, SF-12 MCS was additionally adjusted. When assessing the uptake of flexible working and satisfaction outcomes, both SF-12 MCS and PCS were additionally adjusted.

### Statistical method

We applied the difference-in-difference (DID) method using multivariate linear regressions to identify the average treatment effect on the treated (ATT). The DID method compares the differences in preintervention and postintervention outcomes between the exposure group (which becomes eligible to receive the intervention, ie, the right to request flexible working) and the control group (which does not, in this case, because they already have access to the right to request flexible working). To enhance the robustness of our estimates, we used doubly robust estimation techniques. This approach combines the maximum likelihood estimation of a regression model for the outcome with the inverse probability weighted (IPW) method.^22^ This ensures that our estimators remain consistent if either the outcome model or the IPW approach is correctly specified. The same covariates were included in both the regression model and the IPW calculations (see Covariates).^23^ We calculated the ATT at multiple time points, comparing each time point (wave 6, 8 and 10) to the baseline (wave 4).

## Sensitivity analysis

In order to attach a causal interpretation to DID estimators, researchers routinely invoke the parallel trends assumption that the average outcomes for the exposure and control groups would have followed parallel paths over time in the absence of intervention.^22^ However, the parallel trends assumption is an untestable assumption, so in the results, we also showed the pretreatment effect (by comparing wave 2 with wave 4). Where there were significantly different pretreatment effects, we used HonestDiD in the sensitivity analysis—a robust inference approach to estimate bounds on the post-treatment ATT under varying assumptions about the magnitude of potential violations of the parallel trends assumption.^24^

The uptake of reduced hours arrangements, such as part-time jobs, may depend on the occupation of the employees. A stratified analysis by occupational class was conducted to assess the outcome of reduced hours arrangements.

For the health and satisfaction outcomes, we additionally show the ATT for those who actually use the flexible working arrangements.

## Results

### Uptake of flexible working arrangements

Compared with the control group, individuals in the exposure group are more likely to be men, single, white, under the age of 30 or over the age of 50. The exposure group is also slightly less likely to have a university degree and to be in a professional or managerial occupation (table 1). The standardised mean differences between the control and exposure group after the IPW for baseline characteristics were close to 0, suggesting good balance after IPW (online supplement 3). We also examined that positivity and exchangeability conditions are not violated (online supplement 3).

**Table 1:** Descriptive results of baseline characteristics by control and exposure groups

	Control group (N=8884)	Exposure group (N=8538)	Total (N=15 465)
Age (%)			
<30 years	8.0	25.0	16.3
30–49 years	71.1	33.0	52.4
50–65 years	20.1	38.0	28.9
65+years	0.9	4.0	2.4
Mean (SD)	42.1 (9.2)	43.3 (14.4)	42.7 (12.1)
Gender (%)			
Men	43.8	50.1	46.9
Women	56.2	49.9	53.1
Marital status (%)			
Single	7.8	28.7	18.0
Married	70.0	45.0	57.7
Separated	7.8	10.3	9.0
Cohabiting	14.5	16.1	15.2
Ethnicity (%)			
White	85.1	88.8	86.9
Black	4.4	3.9	4.1
Indian	3.9	2.8	3.3
Pakistani/Bangladeshi	3.8	1.7	2.8
Other Asian/other	2.9	2.9	2.9
Highest qualification (%)			
Degree	33.6	31.0	32.3
Other higher degree	14.3	12.7	13.5
A-level, etc	21.0	23.4	22.2
General Certificate of Secondary Education, etc	21.2	19.7	20.5
Other qualification	6.3	8.0	7.1
No qualification	3.6	5.1	4.3
Occupational class (%)			
Management and professional	47.7	43.9	45.8
Intermediate	16.7	16.7	16.7
Routine	35.6	39.4	37.5
Household income (%)			
Lowest quintile	9.7	6.0	7.9
Second	20.7	11.5	16.2
Third	23.9	19.8	21.9
Fourth	24.9	27.9	26.3
Highest quintile	20.8	35.0	27.7

**Figure 2:**
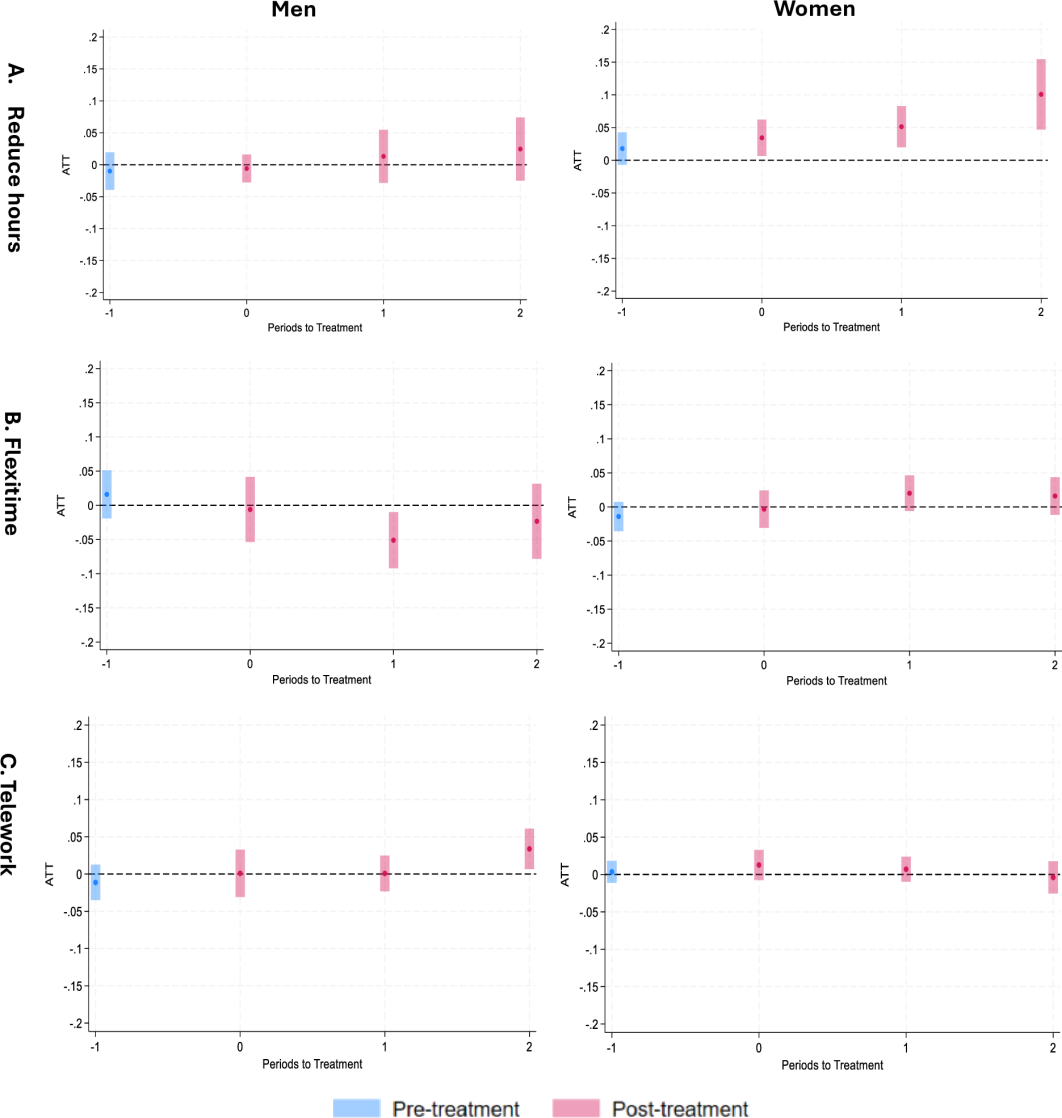
Effect of 2014 policy reform on using reduced hours arrangements, flexitime and telework flexible working arrangements for men (left) and women (right). Note: On the x-axis, time 0 indicates the immediate effect, comparing wave 6 with wave 4 (baseline). Time 1 compares wave 8 with wave 4, and time 2 compares wave 10 with wave 4. Time 1 indicates the pretreatment effect, comparing wave 2 with wave 4. ATT is shown in percentage points. ATT, Average Treatment Effect on the Treated.

Figure 2A illustrates the impact of the policy reform on the use of reduced hours arrangements for men (left) and women (right). The blue bars depict the pretreatment effect, with the 95% CI crossing over zero, indicating no significant pretreatment effect. The pink bars represent the ATT and its 95% CI at various time points. Time 0 indicates the immediate effect, comparing wave 6 with wave 4. Time 1 compares wave 8 with wave 4, and time 2 compares wave 10 with wave 4. The policy reform did not increase the use of reduced hours arrangements for men, as the 95% CI crossed over the zero (dotted horizontal line). Among women, at time 0, the ATT was approximately 0.03. This suggests that the policy reform increased the use of reduced hours arrangements by 3% more in the exposure group compared with the control group, with the impact of the policy reform strengthening over time. By time 1, the effect increased to about 5% and increased to 10% by time 2. Exact values of ATT, p values and 95% CI are shown in online supplement 4. Stratified analysis by occupational class reveals that the increased use of reduced hours arrangements for women was observed among those in management and professional or intermediate occupations, but not among those in routine occupations (online supplement 5).

Figure 2B shows the effect of the 2014 policy reform on the use of flexitime arrangements for men (left) and women (right). For both men and women, the ATT was mainly around 0 at most time points, suggesting no strong effect of the policy reform on the use of flexitime arrangements. The only exception was at time 1, which saw a clear decrease in the use of flexitime arrangements for men, while a slight increase was seen for women.

The effect of the policy reform on the use of teleworking arrangements (figure 2C) was weak, with most ATTs around 0, and a slight increase for men in time 2 only.

### Mental and physical health

**Figure 3:**
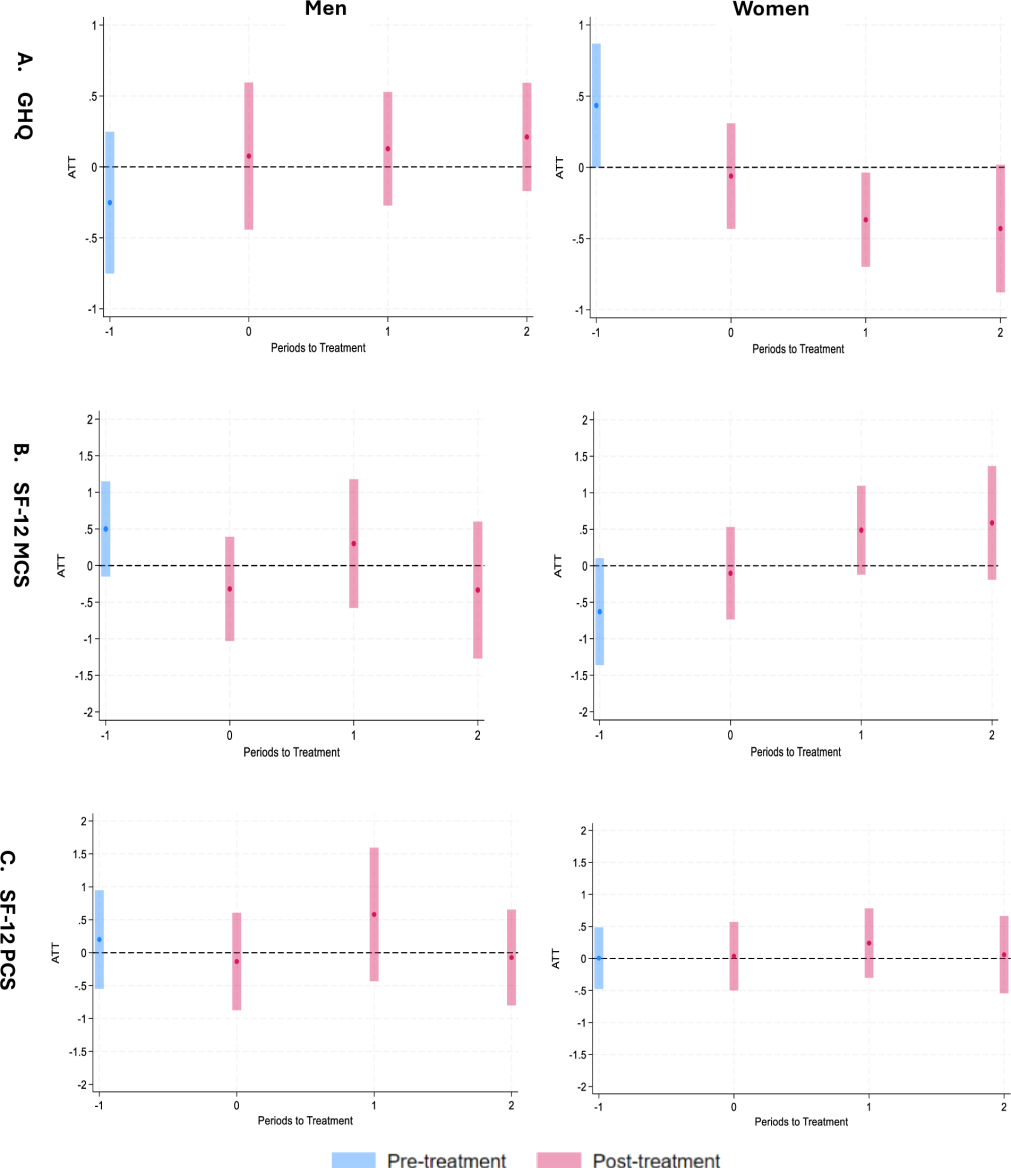
Effect of 2014 policy reform on GHQ, SF-12 MCS and SF-12 PCS for men (left) and women (right). Note: On the x-axis, time 0 indicates the immediate effect, comparing wave 6 with wave 4 (baseline). Time 1 compares wave 8 with wave 4, and time 2 compares wave 10 with wave 4. Time 1 indicates the pretreatment effect, comparing wave 2 with wave 4. ATT is shown in percentage points. ATT, average treatment effect on the treated; GHQ, General Health Questionnaire; MCS, Mental Component Summary; PCS, Physical Component Summary; SF-12, 12-item Short Form Survey.

Figure 3A shows the effect of the policy reform on GHQ scores for men (left) and women (right). The impact was predominantly observed among women. For women, there was a clear trend of decreasing GHQ scores over time since the implementation of the policy reform. The reduction in GHQ scores for women suggests a decrease in psychological distress. The blue bars depict a potential pretreatment effect, that is, potential violations of the parallel trends assumption for women. A sensitivity analysis using HonestDiD suggests that even if the trends in GHQ scores were diverging somewhat between the treatment and control groups before the policy change, the effect remains plausible (online supplement 6). In line with the results for GHQ, the result for SF-12 MCS also suggests that the policy reform was associated with an increase in mental health functioning for women but not for men (figure 3B). There was no association with SF-12 PCS for either men or women (figure 3C).

### Satisfaction with life, job and leisure

**Figure 4:**
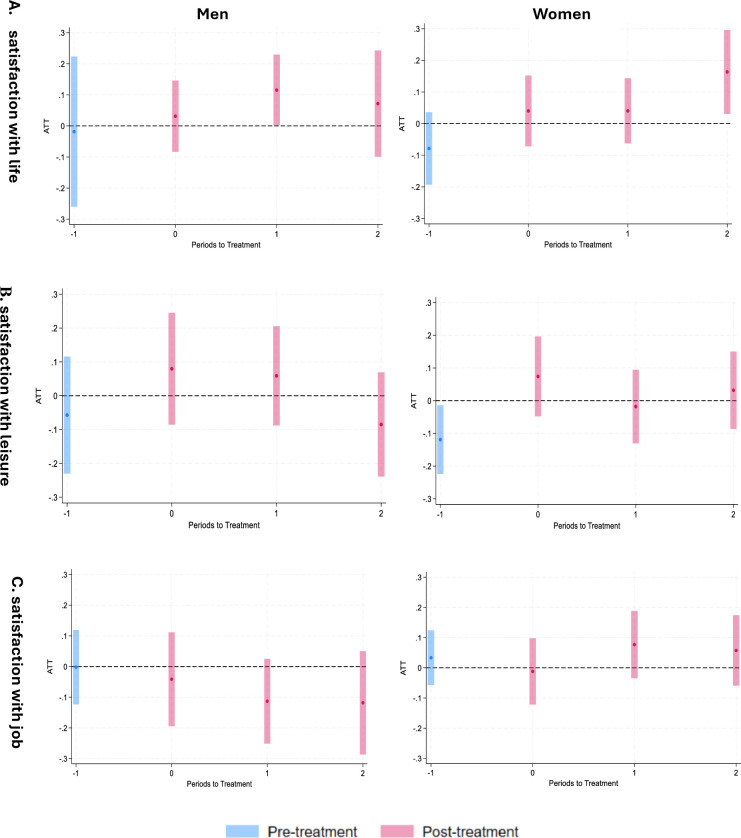
Effect of 2014 policy reform on satisfaction with life, leisure and job for men (left) and women (right). Note: On the x-axis, time 0 indicates the immediate effect, comparing wave 6 with wave 4 (baseline). Time 1 compares wave 8 with wave 4, and time 2 compares wave 10 with wave 4. Time 1 indicates the pretreatment effect, comparing wave 2 with wave 4. ATT is shown in percentage points. ATT, average treatment effect on the treated.

Figure 4A illustrates the effect of the policy reform on life satisfaction for men (left) and women (right). For men, the reform was associated with an increase in life satisfaction of more than 10% at time 1, but there was no significant effect at time 0 or time 2. For women, there was an increase in life satisfaction of more than 15% at time 2. Figure 4 B-C shows the effect of the policy reform on satisfaction with leisure and job satisfaction. No effect was found for either men or women.

Sensitivity analyses among individuals who used flexible working arrangements indicated that effect sizes for certain associations—specifically MCS and life satisfaction—became more pronounced. However, the 95% CI widened considerably due to smaller sample sizes, especially among men (online supplement 7).

## Discussion

Using high-quality longitudinal data in the UK (UKHLS), we examined the influence of the 2014 ‘right to request’ flexible working policy reform. We looked at a wide range of outcomes, including the uptake of different types of flexible working, mental and physical health, life satisfaction, job satisfaction and satisfaction with leisure. Our results suggest that the policy reform increased women’s uptake of reduced hours work arrangements, with the effect growing stronger over time, but did not have the same effect on men. There was no sustained effect on the use of flexitime or teleworking arrangements for either men or women. Additionally, the policy reform resulted in a reduction in psychological distress and improved life satisfaction among women only.

Our findings suggest that the 2014 policy reform, which removed the requirement for caring responsibilities to qualify for the ‘right to request’ flexible working, revealed a gender difference in its impact on individuals’ uptake of flexible working. The reform increased women’s uptake of reduced hours arrangements but not for men. This confirms previous studies suggesting that policies alone do not necessarily allow men access to flexible working arrangements, given gender role assumptions.^25^ Employers may feel more compelled to allow women to reduce their working hours, based on their assumed family responsibilities, and to prevent them from leaving their jobs. In contrast, they might believe that men, as traditional breadwinners, don’t require such arrangements, or that men often receive more support from their partners, enabling them to work longer hours without needing reduced schedules.^26^ Due to fear of negative career consequences, men may not feel comfortable requesting flexible working even when the arrangements were made available via legislative changes.^20^ The 2014 reform increased women’s uptake of reduced hours arrangements but not for other types of flexible arrangements. Despite teleworking and flexible schedules also providing workers with better options to combine work with family responsibilities,^27^ previous evidence shows that there were hesitations from managers to provide workers, especially women, with such arrangements due to existing bias against women’s capacity to work when working from home or working flexible schedules.^28^ It is also possible that employers may prefer reduced-hour arrangements, without significantly reducing manager control of challenging workplace norms. Previous evidence shows that flexitime and teleworking are often viewed with suspicion,^29^ and our findings highlight that national policy alone may not ensure access, particularly the heightened rejection rates for telework and schedule flexibility that challenge traditional workplace norms.^10 25^

We found that this reform improved women’s mental health but not men’s. Similarly, the policy reform improved overall life satisfaction more for women than for men. It is likely that the policy reform on flexible working may have enhanced women’s ability to remain in the labour market while meeting family demands through the reduction of working hours. This may have improved women’s life satisfaction, as they did not have to choose between work or family, and their mental health, as they did not have to work extensive hours to meet both family and work demands.^5^ We do not find any significant association with job satisfaction, as reducing hours would have potentially meant an occupational downgrading for women, although having been able to stay in the labour market.^27^ Despite decades of progress towards gender equality in the workplace and the weakening of traditional work and family roles,^30^ studies consistently show that women still perform the majority of unpaid domestic labour.^31 32^ It is possible that women’s ability to reduce working hours would not have been used to increase their leisure time, but rather devoted to care or housework hours, which explains the insignificant results on satisfaction with leisure. Given that the reform has not largely increased men’s take-up of flexible working arrangements, and even led to a temporary decline in flexitime usage, it is not surprising that the influence of the reform on men’s well-being and life satisfaction was minimal.

The strengths of our study include nationally representative longitudinal data, encompassing a range of well-being measures and various types of flexible working. We applied the DID method, a quasi-experimental method which compares the differences in preintervention and postintervention outcomes between the exposure group and the control group. By following workers for up to 6 years, we assessed both the short-term and long-term effects of the policy change. However, our study also has limitations. Data on flexible working were collected every 2 years, which means some short-term changes in flexible working may not have been captured. Additionally, we lack information on the frequency of flexible working usage, for example, working from home 2 vs 4 days, preventing us from testing whether the policy reform increased the level of flexible working among users. Doubly robust estimators are a relatively new method for estimating the average causal effect of an exposure. As with any new method, caution is warranted.^23^

## Conclusions

The 2014 policy reform resulted in a reduction in women’s psychological distress and improved life satisfaction. This might be partly explained by the increased women’s uptake of reduced hours arrangements, which entail women having control over the number of hours they work to meet family demands. However, no similar increase in uptake was observed among men. Such patterns have the potential to inadvertently exacerbate gender inequalities in labour force participation by pushing women more into part-time work.^10 25 33^ This study highlights the importance of taking gender into account when examining the consequences of flexible working-related policy to avoid intensifying gender inequality in paid and unpaid work.^10^

## Supplementary material

10.1136/jech-2025-224166 online supplemental file 1

## Data Availability

Data are available in a public, open access repository.
