# Supplementary material for: Does the ‘right to request’ flexible work policy influence men’s and women’s uptake of flexible working and well-being: findings from the UK Household Longitudinal Study
Source: J Epidemiol Community Health. 2025 Sep 15;80(1):e224166. doi: 10.1136/jech-2025-224166 (PMC12703338; doi:10.1136/jech-2025-224166)
Supplement: online supplemental file 1 [file jech-80-1-s001.pdf]

Supplement 1 – Literature review of longitudinal studies assessing the effect of flexible working

Search terms were: ((Flexible work*[Title/Abstract]) OR (right to request)) AND (health[Mesh]) AND ((longitudinal) OR (cohort)). We also hand-searched lists of references from relevant papers. We identified three longitudinal studies (Supplement 1). One study assessed the 2003 policy reform that allowed parents of children under six years old to request flexible working. Two other studies did not assess the effect of policy but examined the influence of using flexible working on health. There has been a significant gap in focusing on gender differences and testing both the short and long-term effects of the policy reform.

**Author and year**	**Region, data**	**Study design**	**Results**
Li LZ, Wang S. (2022)	UK, UKHLS	Longitudinal	perceived availability of work-family initiatives improved men and women's mental health by increasing their job satisfaction. Actual usage of work-family initiatives improved women's, but not men's, mental health by increasing their job satisfaction and leisure time satisfaction.
Avendano M, Panico L (2018)	UK, MCS	Longitudinal	The Flexible Working Act in 2003 increased flexible working only among a small group of mothers who had not yet the right to request work flexibility, but it had no impact on their health and well-being
Henke RM et al. (2015)	UK, Prudential Financial employees from 2010 to 2011	Longitudinal	Non-telecommuters were at greater risk for obesity, alcohol abuse, physical inactivity, and tobacco use and were at greater overall risk than at least one of the telecommuting groups.

Supplement 2 – Missing data

Among the eligible sample, most of the missing data comes from missing health outcomes. This is because the health outcomes used in this study were measured in the self-completion questionnaire, which has a much lower response rate than the interviewed questions. Considering the missing data from other covariates is only about 2%, and multiple imputation in combination with Difference-in-Differences method (see Statistical method) is an area of active research interest as to the best approach to take, we have decided not to impute the missing data.

	**Use of flexible working (N)**	**GHQ (N)**	**SF-12 MCS (N)**	**SF-12 PCS (N)**	**Life satisfaction (N)**	**Satisfaction with leisure time (N)**	**Satisfaction with job (N)**
Eligible sample	17,801	17,801	17,801	17,801	17,801	17,801	17,801
After excluding baseline missing demographic covariates†	17,422	17,422	17,422	17,422	17,422	17,422	17,422
After excluding missing baseline health-related covariates*	15,465	15,465	15,465	15,465	15,465	15,465	15,465
After excluding missing outcomes	15,320	15,485	15,465	15,465	15,457	15,457	15,457

† Age, ethnicity, marital status, education qualification, occupational class, working hours, and household income.
* SF-12 MCS and SF-12 PCS

Supplement 3 – standardised mean differences (SMD) of baseline characteristics between control and exposure groups before and after weighting for women and men.

	Women		Men	
	**SMD unweighted**	**SMD weighted**	**SMD unweighted**	**SMD weighted**
**Age**	0.034	–0.005	-0.165	-0.009
**Marital status**				
Single	0.473	-0.027	0.795	-0.234
Married	-0.443	0.009	-0.758	0.145
Separated	0.003	0.005	0.235	0.009
Cohabiting	0.079	0.014	0.009	0.073
**Ethnicity**				
White	0.061	-0.005	0.109	-0.056
Black	-0.040	0.006	0.027	0.022
Indian	-0.064	-0.007	-0.062	0.029
Pakistani/Bangladeshi	-0.050	0.006	-0.201	0.030
Other Asian/other	-0.001	0.004	0.004	0.026
**Highest qualification**				
Degree	0.003	0.005	-0.098	0.030
Other higher degree	-0.081	0.005	-0.010	-0.036
A-level etc	0.037	-0.006	0.092	0.028
GCSE etc	-0.057	-0.003	-0.028	0.021
Other qualification	0.061	0.006	0.025	-0.016
No qualification	0.091	-0.010	0.055	-0.086
**Occupational class**				
Management & Professional	-0.048	-0.002	-0.118	0.021
Intermediate	0.018	0.003	0.005	<0.0001
Routine	0.040	-0.0002	0.103	-0.021
**Household income**				
Lowest quintile	-0.079	-0.017	-0.209	0.045
2nd	-0.243	0.003	-0.254	0.024
3rd	-0.100	-0.004	-0.083	0.015
4th	-0.100	-0.004	0.079	0.021
Highest quintile	0.288	0.005	0.345	-0.077

Positivity assumption: We assessed the distribution of IPWs used in the estimation to ensure that all individuals had a non-zero probability of being either treated or untreated, conditional on covariates.

Summary of Inverse Probability Weights:

- Mean = 2.00, SD = 1.20

- Min = 1.028 (only one case)

- 90th percentile = 3.12

- 95th percentile = 3.94

- 99th percentile = 6.48

- Max = 44.65 (only one case)

The distribution is heavily concentrated between 1 and 4, with very few high-weight outliers. The 99th percentile weight is 6.48, and only a single observation exceeds 20 (Dropping this single observation did not change our results). The mean and standard deviation are 2.00 and 1.20, respectively. This distribution indicates no extreme outliers or mass near zero, suggesting that common support is well maintained across treatment groups and that the positivity assumption is not violated.

Exchangeability assumption: To assess covariate balance after weighting, we computed standardised mean differences for all baseline characteristics. As shown in the Table, all SMDs were below 0.1 after applying IPW, indicating good balance between treatment and control groups. This supports the plausibility of the exchangeability assumption.

Supplement 4 – Effect of 2014 policy reform on each outcome

	Men				Women			
	ATT	P	95%CI		ATT	P	95%CI	
**Reduce hours**								
Time -1	-0.010	0.505	-0.039	0.019	0.018	0.156	-0.007	0.043
Time 0	-0.006	0.598	-0.028	0.016	0.034	0.015	0.007	0.062
Time 1	0.013	0.535	-0.028	0.055	0.051	0.001	0.020	0.083
Time 2	0.025	0.331	-0.025	0.074	0.101	<0.001	0.047	0.154
**Flexitime**								
Time -1	0.016	0.372	-0.019	0.051	-0.014	0.200	-0.035	0.007
Time 0	-0.006	0.803	-0.054	0.041	-0.003	0.820	-0.031	0.024
Time 1	-0.051	0.015	-0.092	-0.010	0.020	0.130	-0.006	0.046
Time 2	-0.023	0.404	-0.078	0.032	0.016	0.252	-0.011	0.044
**Telework**								
Time -1	-0.011	0.354	-0.035	0.013	0.004	0.631	-0.011	0.018
Time 0	0.001	0.950	-0.031	0.033	0.013	0.218	-0.008	0.033
Time 1	0.001	0.943	-0.023	0.025	0.007	0.407	-0.010	0.024
Time 2	0.034	0.014	0.007	0.061	-0.004	0.726	-0.025	0.018
**GHQ**								
Time -1	-0.252	0.323	-0.752	0.248	0.434	0.050	0.000	0.869
Time 0	0.076	0.773	-0.442	0.595	-0.061	0.746	-0.431	0.309
Time 1	0.128	0.530	-0.272	0.529	-0.368	0.029	-0.698	-0.038
Time 2	0.211	0.278	-0.170	0.593	-0.430	0.06	-0.878	0.018
**SF-12 MCS**								
Time -1	0.500	0.131	-0.150	1.149	-0.629	0.092	-1.360	0.103
Time 0	-0.319	0.379	-1.030	0.392	-0.103	0.75	-0.736	0.530
Time 1	0.300	0.503	-0.579	1.178	0.487	0.117	-0.122	1.096
Time 2	-0.334	0.484	-1.269	0.601	0.588	0.139	-0.190	1.366
**SF-12 PCS**								
Time -1	0.200	0.600	-0.548	0.948	0.005	0.984	-0.476	0.486
Time 0	-0.134	0.723	-0.873	0.605	0.036	0.894	-0.498	0.570
Time 1	0.581	0.261	-0.432	1.594	0.240	0.385	-0.301	0.781
Time 2	-0.073	0.844	-0.800	0.654	0.059	0.848	-0.544	0.663
**Satisfaction with life**								
Time -1	-0.018	0.882	-0.260	0.223	-0.079	0.178	-0.193	0.036
Time 0	0.031	0.592	-0.083	0.146	0.040	0.482	-0.072	0.152
Time 1	0.116	0.046	0.002	0.229	0.040	0.444	-0.063	0.143
Time 2	0.072	0.411	-0.099	0.243	0.163	0.016	0.031	0.296
**Satisfaction with leisure**								
Time -1	-0.057	0.517	-0.230	0.116	-0.119	0.026	-0.224	-0.014
Time 0	0.080	0.344	-0.085	0.245	0.074	0.235	-0.048	0.196
Time 1	0.059	0.431	-0.088	0.205	-0.018	0.755	-0.130	0.095
Time 2	-0.085	0.28	-0.239	0.069	0.032	0.598	-0.086	0.150

**Satisfaction with job**								
Time -1	-0.002	0.972	-0.123	0.119	0.033	0.47	-0.057	0.124
Time 0	-0.041	0.597	-0.194	0.112	-0.012	0.833	-0.122	0.098
Time 1	-0.113	0.109	-0.251	0.025	0.077	0.177	-0.035	0.188
Time 2	-0.118	0.169	-0.287	0.050	0.058	0.333	-0.059	0.174

Supplement 5 – Effect of 2014 policy reform on using reduced hours arrangements by occupational class.

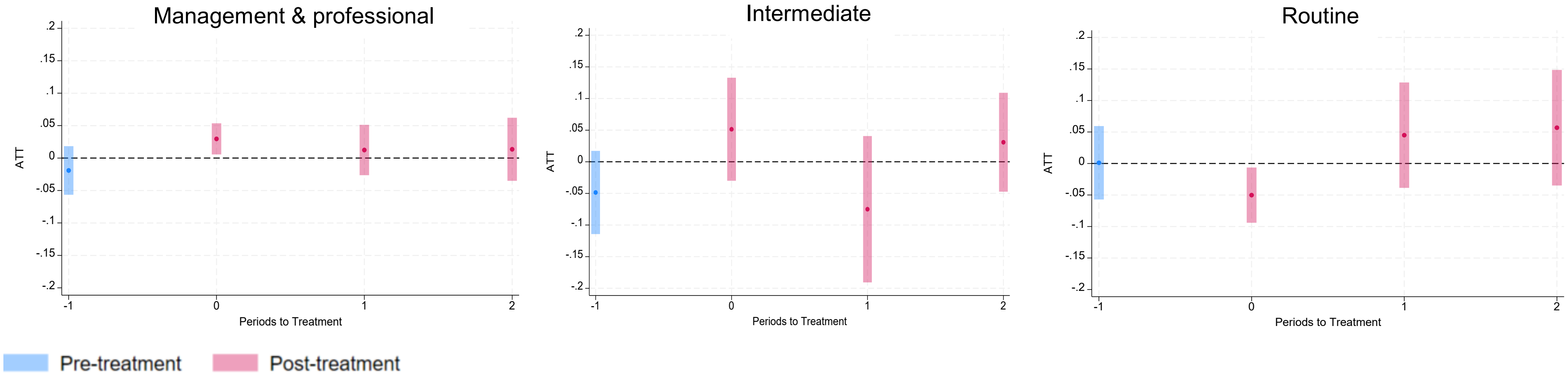

Fig S5.1 Effect of 2014 policy reform on using reduced hours arrangements for men by occupational class.
Note: On the x axis, time 0 indicates the immediate effect, comparing wave 6 with wave 4 (baseline). Time 1 compares wave 8 with wave 4, and Time 2 compares wave 10 with wave 4. Time 1 indicates the pre-treatment effect, comparing wave 2 with wave 4.

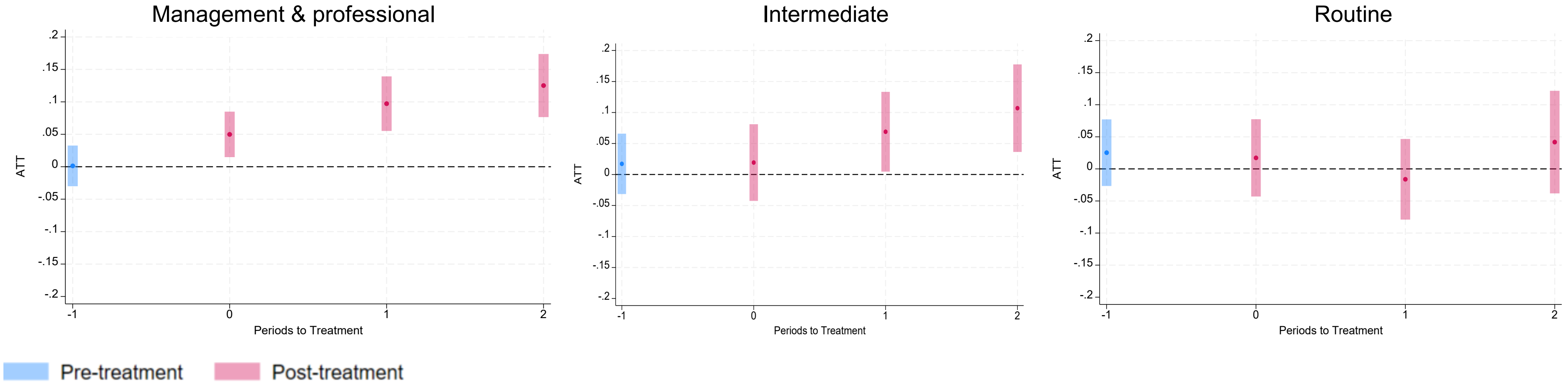

Fig S5.2 Effect of 2014 policy reform on using reduced hours arrangements for women by occupational class.
Note: On the x axis, time 0 indicates the immediate effect, comparing wave 6 with wave 4 (baseline). Time 1 compares wave 8 with wave 4, and Time 2 compares wave 10 with wave 4. Time 1 indicates the pre-treatment effect, comparing wave 2 with wave 4.

Supplement 6– Sensitivity analysis from HonestDiD

A key assumption underlying the difference-in-differences (DiD) design is the parallel trends assumption, which posits that, in the absence of the 2014 policy reform, the trends in GHQ scores for the treatment and control groups would have remained parallel. While the event study plot (right panel of Figure 5) did not show statistically significant pre-treatment differences, we observed some visual divergence that may raise concerns about the parallel trends assumption. To address the possibility of minor violations of the parallel trends assumption, we conducted a sensitivity analysis using the HonestDiD approach (Rambachan and Roth, 2023) to assess the robustness of the ATT for post-treatment outcomes. HonestDiD provides bounds on the estimated post-treatment ATT under varying assumptions about the magnitude of potential violations of the parallel trends assumption in the pre-intervention period. This maximum allowable deviation is represented by the parameter `M`. A larger value of `M` allows for greater deviations from parallel trends and leads to wider confidence intervals.

We performed the sensitivity analysis for women's GHQ scores. We used the `honestdid` command in Stata and employed the `relative magnitudes` method to generate the confidence intervals. We assessed a range of `M` values from 0.1 to 0.5, in increments of 0.1. This range was chosen based on a visual inspection of the pre-treatment trends in the event study plot, as well as considering the scale of the GHQ score.

Table S6 below presents the lower and upper bounds of the 95% confidence intervals for the treatment effect under different `M` values. Fig S6 visualises these results as a coefficient plot. The leftmost point and interval in Fig S6 represent the original DiD estimate and its 95% confidence interval (effectively, `M` = 0). The subsequent points and intervals show how the confidence interval changes as we allow for increasingly larger deviations from parallel trends (increasing `M`).

As clearly shown in Fig S6, the confidence intervals widen as `M` increases, with the upper bound remaining consistently above zero and the lower bound consistently below zero. Crucially, because the confidence interval already includes zero in the original DiD estimate (`M` = 0) and continues to do so across the entire range of `M` values considered, our finding is robust to moderate violations of the parallel trends assumption. Even if the trends in GHQ scores were diverging somewhat between the treatment and control groups before the policy change, the estimated post-treatment ATT remains bounded away from large negative values, and the effect remains plausible.

Table S6. Sensitivity analysis for women's GHQ using HonestDiD

M	95%CI	
.	-0.000 0.869 (Original)	
0.1000	-0.057 0.926	
0.2000	-0.119 0.988	
0.3000	-0.193 1.061	
0.4000	-0.277 1.146	
0.5000	-0.364 1.233	
(method =	C-LF, Delta = DeltaRM, alpha =	0.050)

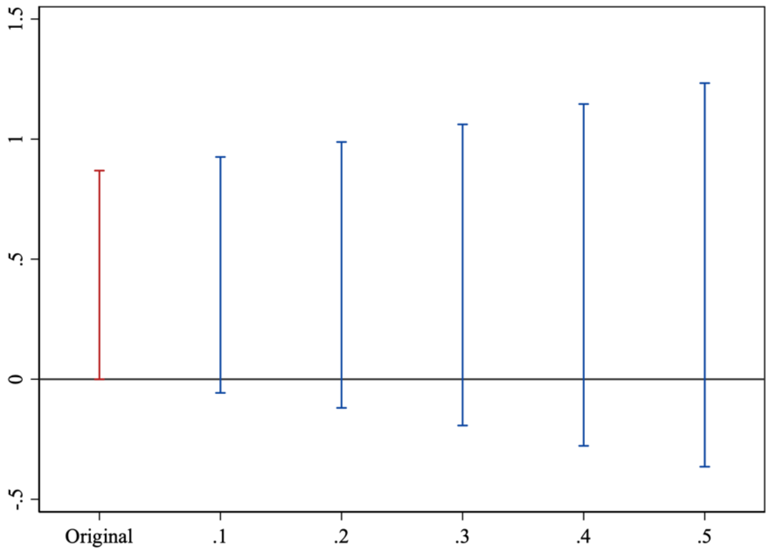

Fig S6. Sensitivity of estimated treatment effect on women's GHQ scores to violations of the parallel trends assumption.

The figure shows 95% confidence intervals for the post-treatment ATT under different values of M, the maximum relative deviation from parallel trends. The horizontal axis represents the M value, and the vertical axis represents the estimated treatment effect on GHQ scores.

Supplement 7-Sensitivity analyses among individuals who utilised flexible working arrangements.

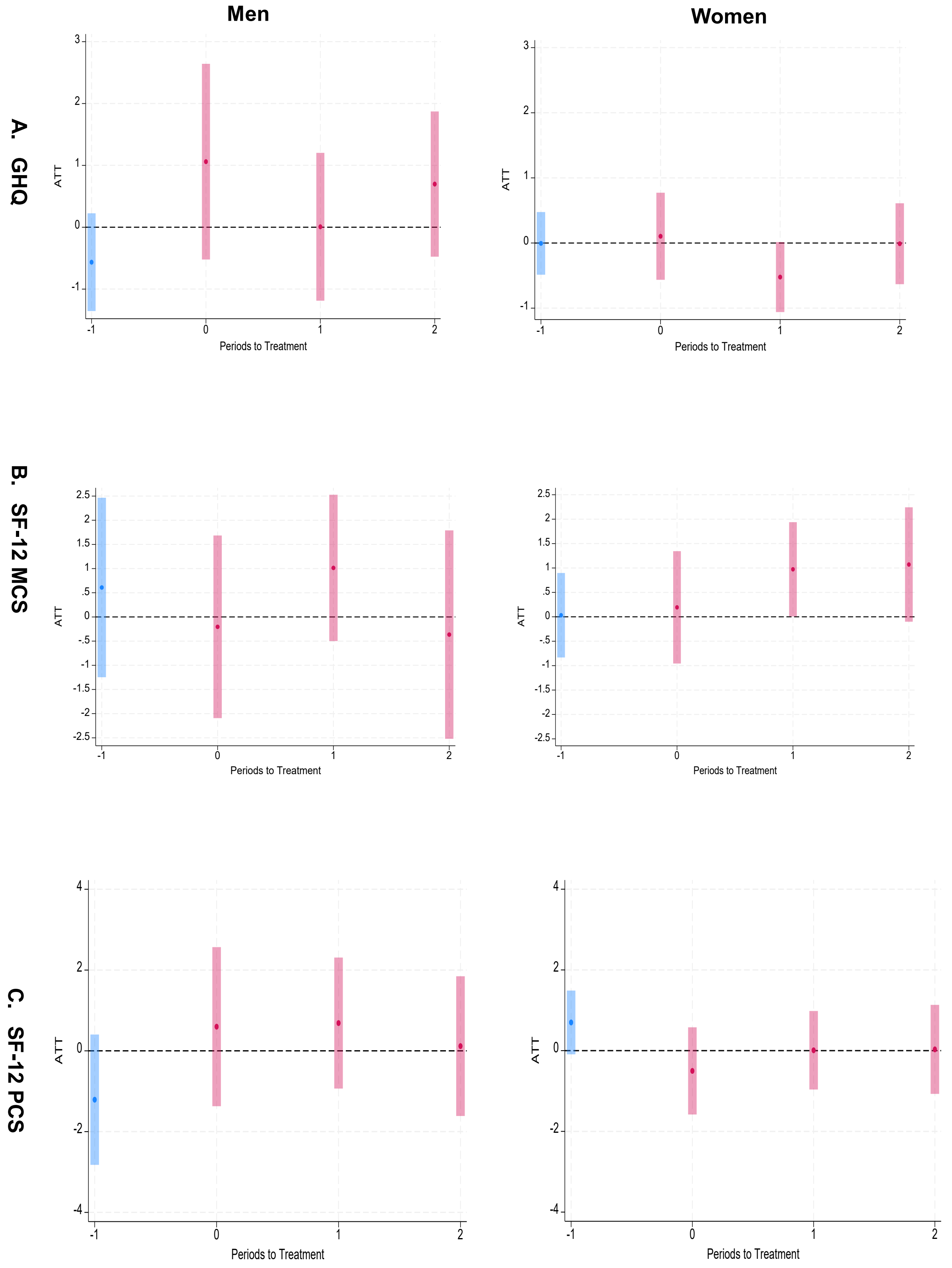

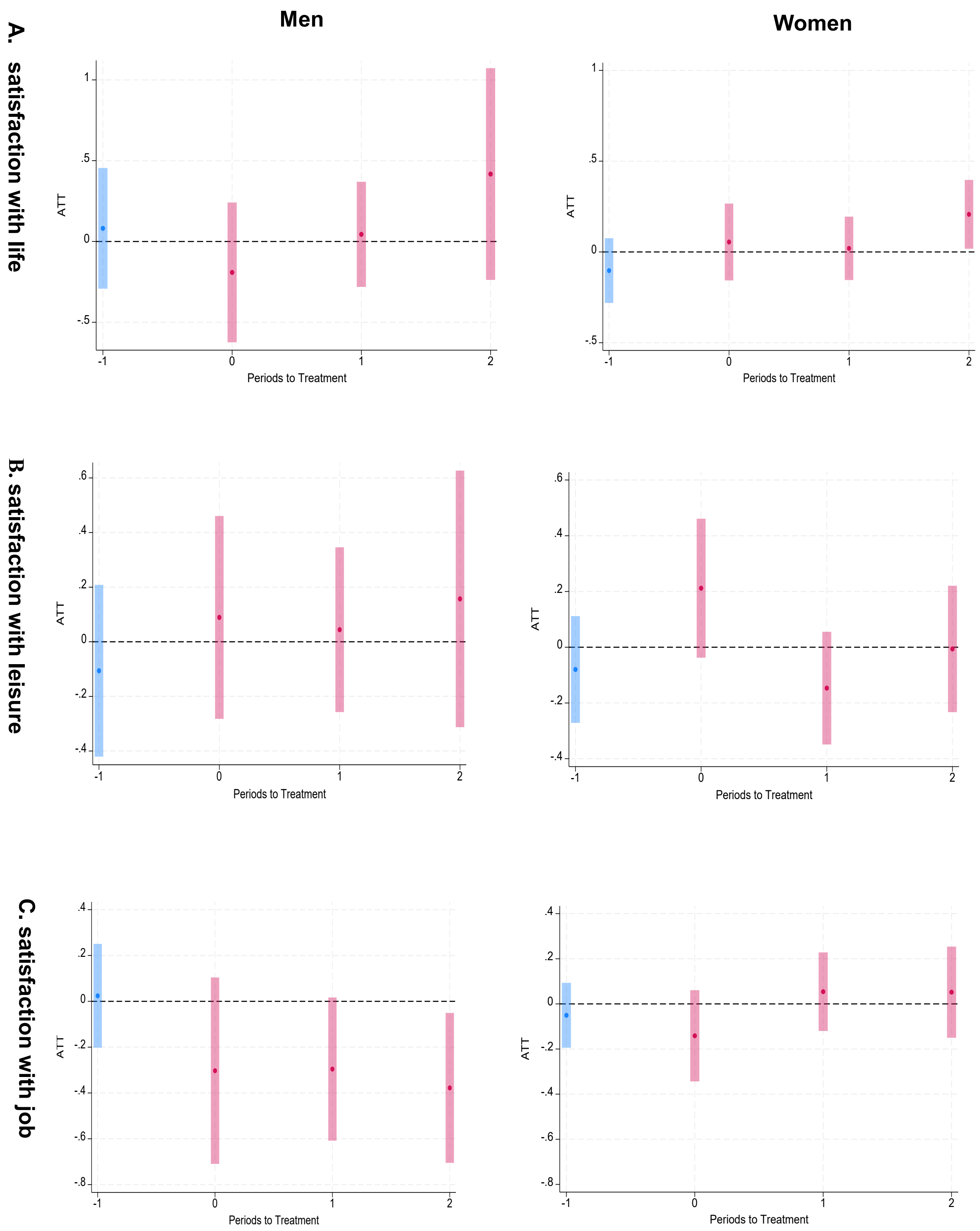

Men
Women
A. satisfaction with life
B. satisfaction with leisure
C. satisfaction with job
ATT
Periods to Treatment
-1
0
1
2
